# A multi‐core ready discrete element method with triangles using dynamically adaptive multiscale grids

**DOI:** 10.1002/cpe.4935

**Published:** 2018-08-31

**Authors:** Konstantinos Krestenitis, Tobias Weinzierl

**Affiliations:** ^1^ Department of Computer Science Durham University Durham UK

**Keywords:** computational geometry, discrete element method, dynamically adaptive cartesian grids, shared memory parallelisation, vectorisation

## Abstract

The simulation of vast numbers of rigid bodies of non‐analytical shapes and of tremendously different sizes that collide with each other is computationally challenging. A bottleneck is the identification of all particle contact points per time step. We propose a tree‐based multilevel meta data structure to administer the particles. The data structure plus a purpose‐made tree traversal identifying the contact points introduce concurrency to the particle comparisons, whilst they keep the absolute number of particle‐to‐particle comparisons low. Furthermore, a novel adaptivity criterion allows explicit time stepping to work with comparably large time steps. It optimises both toward low algorithmic complexity per time step and low numbers of time steps. We study three different parallelisation strategies exploiting our traversal's concurrency. The fusion of two of them yields promising speedups once we rely on maximally asynchronous task‐based realisations. Our work shows that new computer architecture can push the boundary of rigid particle computability, yet if and only if the right data structures and data processing schemes are chosen.

## 1 INTRODUCTION

Granular flows are subject of computational studies in many application fields such as soil assessment in agriculture, powder mixture in engineering or the stability analysis of rocky slopes, and ice sheets. They describe the medium of interest as a set of rigid bodies, which interact with each other through collisions, ie, through contacts. The expressiveness of a simulation of such media is determined on the one hand by the accuracy of the physical interaction model. On the other hand, it is determined by the “economy of scale.” Since statements in these application fields often are statistical trends and as most realistic representations of real‐world setups comprise enormous particle numbers, upscaling the particle count is key. The more rigid bodies (particles) can be simulated the better the prediction on mixing behaviour, the stability of structures, and so forth.

Discrete Element Method (DEM) models study such many‐particle systems. For this, they discretise time and consider two DEM particles to interact with each other if their distance underruns a given threshold, ie, when they are very close to each other. Exact contact is difficult to realise due to the discretised time and, in general, the numerical treatment of the challenge. Identifying contacts is amongst the computationally most challenging steps in DEM^1^, ^2^, ^3^ (reporting from 31 to 34%, 80% or even up to 90% of the whole runtime). Many DEM codes thus stick to explicit single‐step time stepping, analytical particle shapes, often spheres, or composites of these, and furthermore make their primitives have comparable sizes. Finding the closest distance between analytical shapes is computationally feasible as there are analytic distance expressions. This modelling decision however entails a severe abstraction. Most granulates are not analytical or a composition of simple primitives. Furthermore, many materials comprise particles of drastically different size. Whilst many codes stick to simplified geometric primitives such as a sphere, they modify the collision forces. They tailor the forces to make them comprise both first‐principle collision terms (“real collisions”) and terms that imitate non‐spherical behaviour. Many friction or rotation effects, eg, do not arise for real spheres, but can manually be added to a force term and thus make a sphere behave non‐spherical/analytical.^4^ With these modelling decisions, assemblies of (almost) uniform analytical shapes start to realistically model larger structures. Still, many authors doubt that discrete particle assemblies that are not of homogeneous sizes, without sharp edges, and reasonably formed can be represented realistically with analytical shapes,^3^, ^5^, ^6^ despite sophisticated force models. Advance in the field of non‐spherical models, in turn, is restrained by its high compute requirements.^6^, ^7^

Two computer evolution trends suggest that the handling of non‐analytical particles of drastically inhomogeneous size might become feasible soon. Vector registers per core have widened, and the number of cores per compute node is on the rise. Both trends do not directly translate into faster code but require careful algorithm and implementation design. SIMD (Single Instruction Multiple Data) facilities such as AVX (Advanced Vector Extensions), SSE (Streaming SIMD Extensions), and alike help to reduce the compute time of the geometric routines if and only if we design all data structures and data access patterns toward vector processing and accept that data accesses per se are expensive.^8^ We refer to our previous work.^9^ Whilst the vectorisation therein is efficient and the comparison of two particles is parallelised, shared memory parallelisation and data movement minimisation of whole simulation runs pose additional challenges. Compute node counts increase also. Still, the major growth in performance today stems from an increase of core‐per‐node counts and wider registers. The number of compute nodes in supercomputers often stagnates or grows slowly. Multi‐node parallelisation is successfully used in many classic DEM codes.^1^, ^3^, ^7^, ^10^, ^11^, ^12^, ^13^, ^14^, ^15^, ^16^, ^17^ As the particles interact only with local neighbours and typically are subject to small time steps, data exchange is localised and load characteristics do not change rapidly.The present paper does not make a contribution toward inter‐node (Message Passing Interface (MPI)) parallelism or efficiency, but it introduces lightweight data decomposition based upon tasks. These concepts also translate into the shared memory world.

Searches for collision partners typically rely on environments around the particles to reduce the comparison cost from $O(|P{|}^{2})$ to linear complexity. P denotes the particle set. Through the volumetric nature of particles, ie, particles can not cluster arbitrarily dense, we can construct algorithms with linear complexity in |P| for the contact detection that identify all contact points reliably once a reasonable range of these environments is identified. It is however not clear what a good meta/search data structure of minimal extent for the range queries looks like if the particles are of extremely varying sizes and shapes, whilst the meaning of “reasonable range” is not obvious either. Still, the range's extent and the data structure's efficiency determine the constant within the linear complexity. For distributed memory, it is furthermore this maximum search environment that dictates the size of halo regions for which data has to be kept consistent between different MPI ranks. It determines the communication demands. Not only has the comparison cardinality to be small, any data structure design has to facilitate light‐weight shared memory parallelisation intrinsically such that we can scale and balance amongst many cores. Finally, any data structure has to support particles of differing speed and shape. A helper/meta data structure has to identify all potential collision partners per time step. With the predominance of explicit time stepping in DEM,^18^ it however should not constrain the admissible maximum time step size too rigourously.

In the present manuscript, we first propose to use multilevel grids to organise the particles. Particles are binned according to their size, ie, we sort all particles into sets of particles of similar size. All particles of one bin are administered within one well‐suited Cartesian grid. It is as coarse as possible, yet just fine enough to identify all particle‐to‐particle interactions on this grid if any particle compares itself solely with particles that reside in the same grid cell or a neighbouring cell. For the next set of smaller particles, we embed a locally adaptive finer grid into the first grid, on which we treat the smaller particles. We continue recursively. Inter‐grid transfer operators keep particle comparisons of different grid levels consistent, whilst we clarify that the administration of particle‐grid relations, notably the binning, can be done on‐the‐fly without excessive administration overhead. Different to static multi‐resolution grids, our approach employs a dynamically adaptive grid and allows particles to travel between the levels through a lift and drop mechanism. We weaken the one‐bin‐one‐grid constraint. This preserves the conceptual simplicity of regular grids, whilst it minimises the number of grid entities used as well as the number of comparisons to be made for inhomogeneous particle sizes. Quadtrees and octrees are the most popular data structures well‐suited for our particle organisation algorithm. Whilst our experiments rely on a particular spacetree, we refer to any generalisation of the quadtree concept as spacetree,^19^ ie, based upon equidistant three‐partitioning, all proposed techniques apply to any spacetree including quadtrees and octrees, as well as block‐structured grids^20^ that embed grids into each other.

Second, we propose a novel adaptive mesh refinement criterion, which makes the mesh anticipate both the particles' sizes and their arrangement. It is reluctant, ie, tries to work with as coarse meshes as possible without compromising too much on the number of particle comparisons. The coarse grids produced by this novel scheme ensure that we can use bigger time step sizes than naïve adaptive mesh refinement.

Third, we discuss how the multiscale data structure used to administer the particles impacts the shared memory concurrency of a DEM simulation. We identify three layers of parallelism and clarify that none of these layers alone is able to exploit massively parallel shared memory systems. Combined, they however are powerful if we break up the fixed assignment of one time step to exactly one grid sweep, and if we use tasks to realise the collision checks.

Multiscale grids to administer DEM are a known technique.^2^, ^21^ They are conceptually close to established techniques in molecular dynamics^22^ where Lennard Jones‐type potentials enforce particle repulsion weakly. There is however no explicitly preserved molecule volume. Multiscale grids are also well‐known from Smoothed Particle Hydrodynamics (SPH) with short‐range interaction potentials where “particles” completely lack the notion of volume as they describe densities. Our work translates multiscale concepts into the DEM world. Furthermore, it makes three major methodological contributions toward the multiscale DEM paradigm. It formalises the multiscale grid usage and its traversal such that every piece of data is read only once (single‐touch semantics) and the comparison cardinality is linear. It contributes a novel dynamic adaptivity criterion which allows us to run simulations with big time steps. It systematically explores three different types of DEM parallelisation and identifies which types are promising for upcoming machine generations if realised properly. To the best of our knowledge, this triad of methodological contributions and studies is new. It has major large‐scale potential. Whilst our manuscript sticks to single node tests, ie, this is the level where we expect the major growth in performance to happen, our multiscale neighbourhood checks translate into the distributed memory world also. Not a single halo layer (copy of remotely handled particles) is required, but a cascade of layers. Each layer however holds very few particles and the total data transfer is low, compared to a single‐level data organisation hosting particles of massively differing size. Whilst our manuscript sticks to plain explicit time stepping, enlarged cells will massively speed up local time stepping approaches or implicit schemes where all particle movements are not that much constrained by the grid anymore. They suffer less from the stiffness of the underlying problem. Whilst our manuscript sticks to benchmark problems, all advantages demonstrated for toy problems become even more significant once we scale the particle count or details up. They also gain impact when we scale the machine up.

The remainder of this paper is organised as follows. We quickly revise the algorithmic core ingredients of our DEM code in Section 3, before we introduce the multiscale grid employed (Section 4). The main methodological contribution is the discussion of spatial adaptivity and time stepping constraints (Section 5), which interfere with the layers of parallelism from Section 6. Some experiments highlight our achievements as well as open questions before we close the discussion with a brief summary and an outlook.

## 2 RELATED ALGORITHMIC CONCEPTS

DEM or related codes supporting arbitrary shape sizes are rare, and many papers omit runtime impact discussions.^7^, ^23^, ^24^, ^25^, ^26^ Notably, there is no mainstream code that supports arbitrarily triangulated particles, which even can be concave (as in the work of Williams and O'Connor^27^). Instead, most codes model complex geometries via assemblies (composites) of simpler/convex primitives.^7^, ^14^

If we check which particles might collide with which other particles, inhomogeneous particle sizes pose challenges, as one particle might have to be compared to many other potential collision partners, ie, the bigger the area a particle covers, the bigger the area we have to search for potential collision partners. This imposes significant throughput needs, which might not necessarily translate into high computational load.^28^ The state‐of‐the‐art approach is to rely on a regular grid to check a particle within a cell only against particles residing in neighbouring (linked) cells, ie, the regular grid yields the areas to be searched for collision partners. Such a linked‐cell approach can be used as base for Verlet lists^6^, ^22^ or more sophisticated check volumes.^28^ Despite the fact that regular grids deteriorate for extremely inhomogeneous particle distributions,^27^ the largest particle dominates the chosen mesh size, only Thornton et al^21^ seem to used multiple meshes. Here, each particle is hosted within its “fitting” mesh and meshes, and then are compared to each other. Dynamically adaptive meshes based upon recursive space decomposition also reduce the search area for collision partners. However, as long as particles reside on the finest resolution level,^11^, ^27^, ^29^ the deterioration around large particles is only localised but not eliminated. The combination of adaptivity with a multilevel approach is not found in literature. Studies of the interplay of adaptivity criteria with administrative load and admissible time step sizes are, to the best of our knowledge, not published either.

DEM simulations differ in whether they fix the assignment of particles to compute entities, either real nodes or cores, or decompose the domain. In the latter approach, compute nodes or cores own domain fragments and implicitly all particles residing within this domain.^17^, ^29^, ^30^ Studies on domain decomposition with multiscale, dynamically adaptive grids do, to the best of our knowledge, not exist for DEM. Whilst a parallelisation of force contributions (check of different particle pairs, ie, pair interactions) is convenient in the molecular dynamics community,^28^, ^30^ DEM seems to be dominated by spatial parallelisation, ie, different cells (domain areas) are compared to each other concurrently. Our present paper goes beyond selecting one parallelisation strategy, as it compares spatial decomposition to particle‐pair parallelisation. The latter is equivalent a parallelisation of force contributions in the world of molecular dynamics.

Given the predominance of spatial decomposition schemes, the realisation of MPI parallelisation (logically distributed memory) of DEM is well‐understood.^1^, ^3^, ^7^, ^10^, ^11^, ^12^, ^13^, ^14^, ^15^, ^16^, ^17^ Though many roadmaps predict that the gain of performance in future supercomputers will stem from an increase of (shared memory) cores,^8^ literature on shared memory parallelisation in the DEM context however is rare. Whilst data decomposition translates directly into a shared memory world, manycore architectures typically call for more lightweight parallelisation strategies compared to message passing. This is where the present paper makes a contribution though, in return, our lightweight task‐based parallelisation also impacts distributed memory parallelisation design. Multiple papers^3^, ^17^, ^31^ point out that the realisation of MPI parallelisation is not the big challenge in DEM anymore, but that methods have to be found to minimise communication.^28^ We have to exclude as early as possible that unnecessary collision checks are done. Our dynamically adaptive approach based upon spacetrees serves this purpose also, whilst a data movement minimisation, ie, we strive for single‐touch algorithms where each particle data is read from the main memory per time step only once, implies that particle data is to be exchanged only once through message passing if the present strategies are translated into a shared memory world.In this context, we emphasise that our realisation relies on Peano^32^ for which excellent memory behaviour is validated.^19^ We also emphasise that the particle administration is taken from the work of Weinzierl et al^33^ where further communication‐avoiding idioms are introduced. All paradigms proposed here however apply to any octree, quadtree, forest, or spacetree data structure.

## 3 ALGORITHMIC CORE COMPONENTS

DEM with global time stepping is conceptionally simple. The code steps through time loops with a prescribed yet probably changing time step size Δ*t*. Within each time step, the code identifies which particles collide at which locations. Collisions induce forces over the time span which in turn change the angular and translational velocities of the affected particles. The velocities finally determine the particles' subsequent position and orientation. Our sketch sticks to single step methods. Leap frogging, other higher order, multistep, or implicit methods use the same algorithmic ingredients, though those schemes relying on substeps (eg, Runge Kutta) or iterative solves evaluate the collision checks multiple times before they commit the final time integration. Our discussion hence focuses on explicit Euler to uncover the inherent computational challenges.

Particles are rigid triangulated objects in our code. We neither impose any restrictions on the shape of the triangles nor on their arrangement. They can be arbitrary dilated, and the particles can be concave or even toroid, ie, have holes in them. Whilst all particles are rigid and thus may not penetrate, two particles are considered to be in contact if their minimum distance underruns a prescribed threshold 2*ε*. Such an *ε*‐formalism can be read as rigid particles with a surrounding soft (halo) layer of depth *ε*. The formalism resembles Minkowsi sums.^34^ Contact then is equal to a penetration of the halo layers.

Per contact, there is a shortest line between the two bodies. The line's midpoint is the contact point. Any contact point thus falls into the *ϵ* environment of the involved particles, and it is equipped with an outer normal vector *n*. Technically, we represent each contact point as two points with opposite normal directions. The normal vector's length is the distance from the contact point to the surface of the neighbouring particle. Its direction depends on the point of view, ie, from which particle do we look at the contact point. We trivially have |*n*| ≤ *ε*, where *ε* − |*n*| is the (halo layer) penetration depth.

The penetration depth determines the force arising from a contact. Its spatial position relative to the particles' centres of mass clarifies whether the contact induces rotation or translation or both. In the present experiments, we rely on spring‐dashpot's force model with pseudo‐elastic damping^35^ between any two particles $p_{i},p_{j}\in P$. On particle *p*_*i*_, it induces the forces

(1) $$\begin{array}{ll} f_{⊥}(p_{i},p_{j})= & \;\;\left\{\begin{array}{cc} S\cdot (\epsilon -|n_{ij}|)+2D\cdot \left(\sqrt{\frac{1.0}{\frac{1.0}{m_{i}}+\frac{1.0}{m_{j}}}}\right)\cdot \left(v_{ij},\frac{n_{ij}}{\left|n_{ij}\right|}\right)\;\; & \text{if}\;(v_{i},v_{j})\leq 0,\;\text{and}\\ 0\; & \text{otherwise} \end{array}\right.\\ f_{\|}(p_{i},p_{j})= & \;l_{i}\times f_{⊥}(p_{i},p_{j}). \end{array}$$

Each contact point moves the particle and makes it rotate through the repulsive force *f*_⊥_. Through its case distinction, it arises only if two particles approach each other. The tangential force *f*_‖_ in return injects friction into the system. (.,.) denotes the Euclidean scalar product, the scalar *D* models damping, and *S* is the spring coefficient. We use *v*_*i* *j*_ as relative collision velocity *v*_*i* *j*_ = *v*_*j*_ − *v*_*i*_. *m*_*i*_ or *m*_*j*_ denote the mass of the particles *p*_*i*_ or *p*_*j*_, respectively, whilst *n*_*i* *j*_ is the contact normal pointing from the contact point in‐between the particles onto the surface of particle *j*. Finally, *l*_*i*_ is the lever arm of *p*_*i*_'s centre of mass to the contact point. Through vector products, it determines both how *f*_⊥_ translates into rotation and the arising friction.

As we study arbitrarily shaped rigid bodies, we might obtain more than one contact point per particle pair. The total forces thus result from a sum of per‐contact contributions.

A naïve implementation of one explicit Euler time step reads as follows.

1. Run over all particle pairs $p_{i},p_{j}\in P$. Run over all triangle pairs *t*_*i*_,*t*_*j*_ from the two particles. If two triangles are closer than 2*ε*, they yield a contact point.
2. Eliminate redundant contact points. Redundant contact points are induced, eg, by any two triangles that are connected through one vertex, which is also the closest point to a neighbouring surface. Each of the two triangles compared to the neighbouring surface then yields this very contact point. Other redundant points arise from whole faces hitting another object, ie, from non‐point collisions. Sophisticated elimination algorithms to identify non‐physical pairs of contact points are known.^25^ For our setups, we however neglect/simplify this application challenge and average any two contact points for a particle into one, if they are closer than the particle's minimum mesh size and carry reasonably close normals.
3. Compute a force per contact point. Accumulate per particle all forces arising from (1).
4. Update the particles' state.

We close our discussion of the core algorithmic ingredients with some observations.

- As we work with rigid particles, particles may not penetrate each other. We thus have to choose a suitably small time step size. Though our system can grow stiff, in general, we want to choose time step sizes as big as possible to speed up the simulation.
- A straightforward algorithm implementation is in $O(|P{|}^{2}\cdot T_{max}^{2})$. P is the set of particles. $T_{max}$ is the maximum number of triangles per particle. This complexity renders the implementation computationally infeasible or at least challenging.
- With large $T_{max}$, ie, a reasonable geometric level of detail, the cost per particle‐to‐particle comparison becomes significant.

## 4 OCTREES AS MULTISCALE LINKED CELL GRIDS

### 4.1 Linked cells

Linked cells as used in molecular dynamics^22^ are a straightforward technique to tackle the quadratic complexity in |P|. Let *r*_*i*_ be the radius of the bounding sphere of a particle *p*_*i*_ and let $r_{max}=\max_{p_{i}\in P}\;r_{i}$ identify the biggest bounding sphere of all particles. We cover the computational domain with a regular Cartesian mesh Ω_*h*_. We decompose it into cubes. Their side length is at least 2(*r*_*m* *a* *x*_ + *ε*). A particle is said to fall into a cell if the centre of its bounding sphere lies in the cell. A particle can collide with another particle if both particles either fall into the same mesh cell or into mesh cells which are at least vertex‐connected. The connectivity property coins the phrase linked cell. Links however are technically not required to be held, as a regular Cartesian grid is topologically trivial, ie, the index of a left, upper, …neighbour cell of any cell can directly be computed. Furthermore, the hosting cell of any particle can be computed from the particle's bounding sphere centre.

There are two paradigms to choose from when we implement this approach. In a first variant, we can treat the grid as helper data structure for the neighbour lookups. Prior to the time step, we create a grid and bucket sort all particles into this grid. The collision detection traverses all particles. Per particle, we determine the particle's grid index, and then we run over all particle indices assigned to this cell plus the cell neighbours. It is convenient to check only half of the neighbours to avoid redundant collision identifications. In a second variant, we treat the grid as owning meta data structure, ie, we make it responsible for the particles. Each cell holds pointers to all particles contained. In this case, the algorithm runs over the cells rather than the particles directly. Per cell, it processes all 3^*d*^ vertex‐connected cells and evaluates all particle pairs induced by the cell pairs. Our code follows the latter paradigm. Again, we exploit symmetries and do not evaluate cell pairs twice.

Orthogonal to the data ownership distinction above, we rely on the dual mesh rather than the cells.^33^ Our code stores each particle within the vertex closest to its centre. Effectively, we then perform all neighbour checks on the dual Cartesian grid. Our code runs over the cells. Whenever we read a vertex in for the first time, we check all particles assigned to this vertex against all other particles of the vertex. Per cell, we compare the particles assigned to its 2^*d*^ vertices if and only if their centres fall into the respective cell. Again, we do not re‐check any two particles assigned to the same vertex and we skip face‐connected vertex pairs that have been studied before.

Time step sizes in DEM are typically small. The particles do not dramatically change their position between any two time steps. It is thus convenient not to build up the meta data structure from scratch per time step. Instead, we preserve the map from grid vertices to particles and only update links for those particles for which the particle‐vertex association has changed due to translation. If the position update suggests that a particle leaves its vertex's surrounding, we re‐assign it to another vertex.

Some observations result immediately.

- If all particles are of the same or very similar size, the complexity of the overall algorithm reduces to
(2) $$O(|P|\cdot T_{max}^{2}).$$
- An upper bound for the global time step size
(3) $$\Delta t\leq \frac{2(r_{max}+\varepsilon )}{v_{max}}$$
  results from the fact that no particle may move more than one cell at a time. Our vertex‐to‐particle mapping otherwise would become inconsistent, and our neighbour collision searches might fail. *v*_*m* *a* *x*_ is the maximum speed of all particles in the system.

Our observations document the two problems linked cells on regular grids do face, ie, (i) though the particle size distribution might be (very) inhomogeneous, only the biggest *r*_*m* *a* *x*_ determines which mesh size we are allowed to use. If lots of very small plus few big particles are used, large sets of small particles might cluster within the cells. The search for neighbours remains linear in |P|, but the linear complexity then comprises a large constant. We might have to check a bounded yet large number of particles per cell. In the (theoretical) limit, ie, for smaller and smaller particles suspended in‐between the big ones, the constant can grow into the order of |P|. In a worst‐case scenario, all small particles, ie, all particles from P besides the few big ones, collocate in one cell. Equation (2) degenerates. (ii) If the particle mass or energy distribution is (very) inhomogeneous, there will be some, typically very small, particles that determine *v*_*m* *a* *x*_ and thus determine Δ*t*. The solver then requires many tiny time steps as the system stiffens.

### 4.2 Two‐scale linked cells

Let *h* ≥ 2(*r*_*m* *a* *x*_ + *ε*) be the mesh size of a grid Ω_*h*_. Let there furthermore be a second grid Ω_*h*/*k*_,*k* ≥ 2 with a mesh size that is a fraction of *h*. We use *k* = 3 here as we make our work use.^19^ Still, any *k* ∈ {2,3,…} is admissible. We enforce $\Omega_{h}\subset \Omega_{h/k}$, ie, the finer grid embeds into the coarser grid. We however hold them separate. Per grid, we distinguish two types of particles, ie, “Normal,” real particles are assigned to one grid vertex each. Their bounding sphere's diameter 2*r* plus twice the *ϵ* radius has to be smaller than the respective grid's mesh spacing. Virtual particles in contrast are copies (realised as pointers) from a coarser mesh. They are associated to (multiple) grid vertices and their diameter typically exceeds the mesh size. Because of these conventions, the coarser grid does not hold any virtual particles in a two‐grid system.

**Figure 1:**
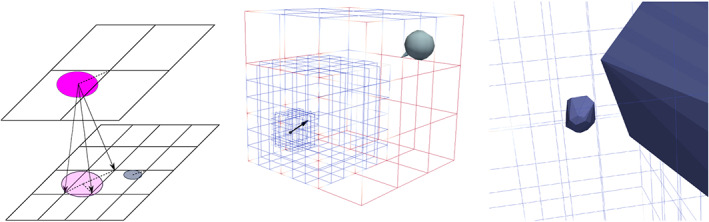
Left: two regular grids are embedded into each other. Each level holds one particle fitting into its mesh width. Real particles are associated to the vertex nearest to their centre. Coarse particles are held as virtual particles on the finer grids (links). Three of these inheritances are visualised here. Middle: two particles approach each other and the adaptive grid follows them. Right: zoom into constellation just before the particles collide

We introduce three operators on our two meshes. First, drop accepts a particle with radius *r*_*i*_ on mesh Ω_*h*_. If 2(*r*_*i*_ + *ε*) ≤ *h*/*k*, the particle is removed from its host mesh and assigned to the vertex in Ω_*h*/*k*_ that is closest to the particle's centre. We use drop also on a whole mesh, which means that all particles of a mesh are subject of the checks and, if admissible, are dropped. drop sieves particles through the coarser mesh.^36^ It realises the particle size binning from the introduction. Second, lift takes a particular particle on Ω_*h*/*k*_ and assigns it to the vertex in Ω_*h*_ that is closest to its centre. lift is the counterpart of drop. Both lift and drop are defined on real particles only. Finally, project takes a particle from Ω_*h*_ and creates virtual particles in Ω_*h*/*k*_ as follows: Every vertex in Ω_*h*/*k*_ holds a virtual particle, ie, a pointer to the original one, if it falls into a sphere around the particle with radius $r_{i}+\varepsilon +\frac{h\sqrt{d}}{k}$. *r*_*i*_ is the particle's bounding sphere radius. To increase the robustness (for adaptive grids), we typically employ the more conservative formula $r_{i}+2\varepsilon +\frac{h\sqrt{d}}{k}$. Virtual particles hence are not always associated to the vertex closest to their centre, and multiple virtual particles representing one real particle may exist on a particular mesh level (Figure 1).

For two grids, only non‐virtual particles on Ω_*h*_ induce virtual particles on the next finer mesh as Ω_*h*_ does not host virtual particles by definition.

We use project also on a whole mesh, which again runs over all real and virtual particles of this mesh.

After the initialisation of our DEM simulation, we run drop. This ensures each particle that fits to mesh Ω_*h*/*k*_ is hosted on this mesh. The other particles remain on the coarser one. We bin the particles according to their size. After each time step, we eliminate all virtual particles from the grids and then run project. This establishes links in Ω_*h*/*k*_ to particles in Ω_*h*_. Our code now traverses both meshes. Per vertex, all particles are compared to all particles plus all virtual particles. Per cell, all real particles that are assigned to the 2^*d*^ vertices of the cell are compared to all real and virtual particles assigned to the other 2^*d*^ − 1 vertices. Again, symmetries are exploited to avoid duplicate comparisons, and real particles are subject to checks if and only if their centre coincides with the respective cell.

This realisation augments the linked cell algorithm with two‐scale operators through the virtual particles. It recursively extends to more than two grids. We note that our approach relies on the grid having valid links to the particles at all times. It then becomes a classic bottom‐up collision detection where collisions of small particles with huge particles are identified on the finer mesh only. We conclude that our approach studies fine‐to‐coarse particle relations, which typically are of smaller granularity than coarse‐to‐fine relations.^21^ This observation implies non‐rigorous bounds on the computational cost per grid entity pair.

### 4.3 Spacetrees as locally adaptive multiscale linked cell data structure

Using regular multiscale grids plus grid traversals to identify particle collisions is computationally inappropriate if particles cluster or small particles squeeze homogeneously into assemblies of bigger particles. In both cases, levels tend to be sparsely populated. Traversing all of their cells induces overhead. Spacetrees^19^ tackle this drawback as they span cascades of regular, yet ragged Cartesian grids. A spacetree starts from one square (*d* = 2) or cube (*d* = 3), respectively, which covers the whole computational domain. We call this cell root and cut it equidistantly into *k* pieces along each coordinate axis. *k* = 3 here. Whilst root spans a trivial Cartesian mesh Ω_*h*_ with only one cell and 2^*d*^ vertices, this first construction step yields a new mesh Ω_*h*/*k*_ with *k* ^*d*^ cells. The two meshes fit into each other and thus fit to all grid properties exploited in the previous section. As we continue to apply our construction process recursively yet independently per cell, we obtain a cascade of meshes $\Omega_{hk^{-\ell }}$. *ℓ* ≥ 0 is called level. The meshes all fit into each other, yet some of them might not cover the whole domain. The meshes are ragged. As we hold the ragged meshes separately, multiple vertices may spatially coincide. A vertex (plus the particles it hosts) thus is unique through its position plus its level. Furthermore, some vertices can be hanging, ie, can be surrounded by less than 2^*d*^ cells on the same level.

If a particle moves, it either remains assigned to its former “host” vertex or it is assigned to a cell‐connected different vertex. If this new vertex is a hanging vertex, ie, if this destination vertex is not surrounded by 2^*d*^ vertices on the same grid level, we lift the particle to the next coarser level. Our code does not impose any balancing on the tree^37^ and thus particles may rush several levels up in the tree per time step. Whenever a particle's centre moves into a new cell, which overlaps with *k* ^*d*^ cells on the next finer level, we make the particle subject to drop if and only if the vertex a particle would be re‐assigned through drop is not a hanging vertex. Again, drop can be applied recursively, ie, particles may drop several levels in one rush.

How to store and traverse spacetrees efficiently is subject of many publications. Key for the present paper is to run through the tree, ie, to traverse the grid levels element‐wisely, and to do this top down. If a cell is traversed, we take it for granted that the next coarser cell spatially overlapping has already been traversed. This induces a multiscale mesh traversal. Depth‐first (DFS) and breadth‐first (BFS) are the two most popular fitting tree traversals. Both preserve a top‐down ordering. Both rely exactly on the same multilevel operations as used in our two‐grid presentation. In practise, we found DFS an advantageous traversal, as it can be realised as plain recursive function where all multilevel information (which is the next coarser node in the tree and which are its adjacent vertices) for lift and drop is implicitly available through the call stack. Furthermore, DFS in combination with space‐filling curves is well‐known to yield good memory access characteristics.^19^

Whilst we refer to the works of Weinzierl et al^19^, ^33^ for details on the used data management, we elaborate the key finding of a top down traversal for our algorithm. A multiscale element‐wise tree traversal knows at any time which vertices are adjacent to the currently processed cell and whether one of these vertices is processed for the first time, with all non‐hanging vertices being subject to 2^*d*^ “visits”. As particles are associated to vertices, it can issue all particle‐to‐particle checks throughout the grid traversal. A multiscale top‐down tree traversal knows at any time through the call stack what the parent node of the currently processed cell, ie, the next coarser node in the tree, is. It can issue all lift and drop operations throughout the traversal. A multiscale top‐down tree traversal can clear all virtual particle lists on fine vertices when it descends into a subtree. It then can inherit all (virtual) particle information from the coarse grid. Virtual particle lists are not to be stored persistently but can be built up on‐the‐fly throughout the depth‐first traversal. Finally, an element‐wise tree traversal can, on‐the‐fly, maintain all associations of particles to vertices. Every vertex holds a list of its real particles. As particles may not travel more than one cell at a time, the particle will after the move be associated to one of the 2^*d*^ adjacent vertices of the current cell. Whenever one of its particles' centre falls into the respective tree, the element‐wise traversal can reassign to their new host vertex. No additional bookkeeping is required.

### 4.4 A single‐touch DEM realisation

**Figure 2:**
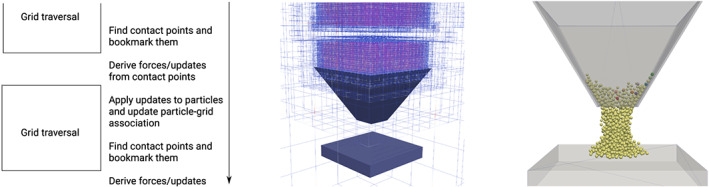
Left: we shift the DEM workflow relative to the grid traversals by half a grid traversal. Middle: a hundred thousand particles on top of the funnel. The particles are drop to the fine levels of the grid, and the grid adopts around them when they fall through the hopper. Right: particles squeezing through a hopper setup. One of our standard validation setups where non‐analytical particle shapes induce distortions in the symmetry of the resulting pile at the bottom

Memory access is expensive in terms of runtime on current architectures. Good algorithm implementations therefore are single‐touch, ie, read each piece of data only once per time step. Reduction of memory and memory access is only one metric of code quality, and it often competes against streaming and vectorisation properties. In the work of Krestenitis et al,^9^ we explicitly introduce data redundancy to facilitate SIMDability and avoid non‐continuous data access. The present manuscript follows these lines and assumes that any large memory footprint is acceptable as long as all data access per particle is continuous (streaming) and the particle as a whole is read from main memory only once per time step. We strive for a single‐particle‐touch approach. To achieve this, we propose to abstract from the idea to realise one DEM time step through one grid sweep. Instead, we shift the actual particle updates by half a grid sweep (Figure 2).

1. We determine particle contact points whenever we run into a cell or into a vertex. These points are bookmarked throughout the traversal.
2. After the traversal has terminated, we determine all particle updates from the collected contact points. Before that, we make them subject to duplicate elimination. The set of contact points is erased afterwards. We store all particle updates in a separate table. They are not applied directly.
3. When we run through the tree the next time, we apply the particle updates as well as lifts and drops prior to the next collision detection. The particle update is a preamble to the next particle update. It dequeues particle updates from the update table, applies them, re‐associates the particles to vertices (if necessary), and then hands over to the subsequent contact detection.

Our contact detection works bottom‐up, ie, fine grid particles are compared to particles on the current level plus the virtual particles on this level which are links to coarser levels. A top down tree traversal thus is mandatory to ensure that all grid and particle data are consistent: It first updates the positions of all particles on this level, ie, all particles associated with coarser levels within the tree are updated already, before it triggers the collision checks on the current level. The overall scheme still realises one compute time step per tree traversal, but it requires one kick off traversal.

The realisation of the single‐touch scheme is straightforward. A DFS variant is given in Algorithm 1. Any single‐touch element‐wise traversal intermixes operations on the different grid levels. Some regions of the tree already might have been subject of collision checks, whilst other regions have not been traversed yet.As the different aspects particle bookkeeping and DEM physics have differing concurrency characteristics, we found it advantageous to realise the position updates through the descends within the tree, whilst we embed the actual physics into the traversal backtracking. Fusing them into the descends only would work also.

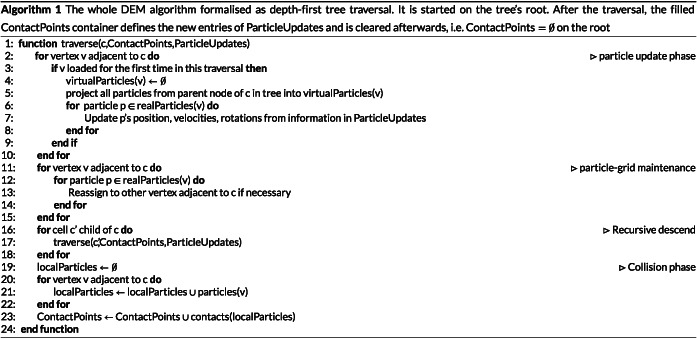

Our complexity claim (3) lacks a term modelling the impact of project, even though project creates virtual replica of a particle on subsequent levels and thus effectively increases |P|. Indeed, very huge particles hosted on coarse grids induce many virtual links on the finer grid levels. However, we (i) reiterate that these links are used if and only if they are mapped onto grid entities which also host real particles. The bookkeeping of virtual links induces some overhead. We assume it to vanish behind the real computational load. No actual data transfer or computation is triggered by project directly. (ii) project is a spatially localised operation evaluated on‐the‐fly for all coarse particles. Coarse particles induce links only on those finer vertices which are close by. The creation of virtual links ripples over the cascades of finer levels in a top down manner, but a lookup of a coarse particle itself within the collision detection does not induce an additional |P|‐dependent cost term. It is an indirect memory access only. (iii) Collision checks within a cell between a fine particle hosted locally and a virtual particle hosted on a coarser grid may induce redundant checks as the coarse particle might be linked from multiple vertices. It is straightforward to eliminate at least the redundant links per cell. (iv) Finally, we discuss strategies how to keep adaptive grids shallow in the follow‐up sections. It is thus convenient to assume that (3) remains valid, though with a larger constant, in the adaptive regime.

We refer to our approach as single‐touch implementation through very large particles might be read multiple times per grid traversal. They are read through their hosting level, and then re‐read on finer grid levels through the virtual links. For an inhomogeneous particle size distribution, we however assume that the very coarse particles form a minority.

## 5 DYNAMICALLY ADAPTIVE MESHING WITH ADAPTIVE GLOBAL TIME STEPPING

### 5.1 Dynamic adaptivity

We propose two different combinations of adaptivity plus coarsening criteria. Our (naïve) *adaptive spacetree* starts from a regular grid spanned by a spacetree which can accommodate even the largest particles. The start‐off grid is determined by *r*_*m* *a* *x*_. Prior to any time step, we refine the 2^*d*^ cells around any vertex where a drop of any of the assigned particles would sieve this particle to the next finer level. The refinement is not realised as separate algorithmic step. It seamlessly integrates into the spacetree traversal. Once all particles within a cell are updated, we evaluate the criterion and dynamically refine the spacetree further. This happens throughout the top‐down steps and is a localised operation that does not further affect the tree traversal logic. It plugs directly between the particle‐grid maintenance and the recursive descend of the traversal.

Our partnering (naïve) *coarsening criterion* relies on the notion of an analysed tree grammar.^38^ Let a vertex *a* be a child vertex of a vertex *b* if any adjacent cell of *a* is constructed from an adjacent cell of *b* through one spacetree construction step. Prior to the tree traversal, each vertex that does not hold a particle is a coarsening candidate. A vertex that is a coarsening candidate remains a coarsening candidate if and only if all children vertices on the next finer level are coarsening candidates. Our coarsening criterion is defined recursively over the tree levels. It plugs into the backtracking of the top down traversal. Once a tree traversal has terminated, we remove a patch of 3^*d*^ cells from the tree if and only if all of their adjacent non‐hanging vertices are coarsening candidates.

Our *reluctant* spacetree refinement criterion refines a cell if there are at least two particles assigned to the cell's 2^*d*^ vertices, if these particles might yield a contact point, and if at least one of them would be affected by a drop. That is, we first check per unrefined cell whether there is at least one particle pair, both virtual and real particles are taken into account, hosted through the cell's 2^*d*^ vertices. The “might yield” circumscribes two computationally cheap checks. For any pair, we determine whether they approach or not and whether their bounding spheres do overlap. Only particle pairs for which both criteria hold are studied further. If one of the two of them would fit into the next finer grid if there were a grid, we refine the tree.

The reluctant adaptivity criterion is accompanied by *aggressive coarsening*. We hold one integer marker per vertex. At the start of a grid sweep, the integer marker is halved, before we re‐add the number of particles associated to the vertex to the marker. On top, we add the marker values from all child vertices. Finally, if a cell refines, all new vertices created are added a further fixed value (typically 4 or 8). A vertex is a coarsening candidate in the sense of the description above if its marker is smaller than a given threshold. We typically use less than half of the value that we add upon refinement. 2 or 4, respectively, seem to be reasonable.

Fine grid regions move along the trajectories of the small particles in our adaptive spacetrees. Once all fine particles have left a subdomain, the grid coarsens. The particles drive the meshing. Whilst such an approach eliminates comparisons induced by too coarse meshes, it tries to minimise the number of particle comparisons actually triggered as the mesh is chosen that fine that really only particles in neighbouring cells are collision candidates, it induces rather detailed meshes with lots of cells. Many of its mesh cells however might be empty, in our tree world, an insertion of one additional cell always triggers *k* ^*d*^ − 1 additional cell creations independent of whether they hold data or not, or hold solely virtual particles and thus do not yield compute load. Traversing empty or very cheap cells is not for free on computer generations where bandwidth is a limited resource. Furthermore, empty cell traversals are hard to parallelise as they have almost no computational load.

Our reluctant grid reduces the number of mesh elements. As long as particles are far away from each other or do not approach, it sticks to meshes that seem to be “too” coarse for the chosen particle size. A comparison of such particles is cheap. Their bounding spheres and approach behaviour act as guard and tell the algorithm right from the start that the particles' triangles are not to be studied. Only once the triangle‐to‐triangle comparisons kick in, it refines the grid further to avoid too many checks in subsequent time steps. The price for the reduced mesh detail is thus a slightly increased comparison count. Our fundamental hypothesis is that the additional comparisons we have to make are computationally cheaper than the effort we would otherwise invest into traversing (almost) empty tree cells. The comparisons drive the mesh adaptivity.

The reluctant's counterpart, the aggressive coarsening criterion, in turn, removes mesh elements even if multiple particles are close. Such an approach tends to introduce mesh oscillations. Mesh parts are coarsened and then immediately refined again. This is disadvantageous as the accompanying lifts and drops are not free. Data is allocated and de‐allocated. We thus use annealing. Vertices hold a (heat) marker that vetoes any coarsening of surrounding cells whilst they are still hot. They are cooled down per time step, and are heated up by associated particles or particles hosted on spatially near particles on finer levels. Finally, they experience heat “shocks” by every refinement which then have to diffuse away before a vertex can become a coarsening candidate again.

Any dynamic grid refinement immediately induces drops of particles. Drops integrate seamlessly into our algorithmic sketch. No additional particle operation is required. If a vertex is destroyed and holds particles, these particles are lifted to the next coarser level prior to the grid destruction. No additional particle administration routines are required by either of our dynamic mesh refinement criteria.

### 5.2 Time step size constraints

Besides the mesh‐induced time step size constraint of (3), we have a second physical constraint to consider: Any global time step size has to be chosen such that no two particles penetrate or even tunnel through each other. At the same time, the time step size should be as big as possible to reduce the time‐to‐solution. We propose the following approach, which we found reasonably robust.

Our *creeping time step increase* reduces the maximum velocity *v*_*m* *a* *x*_ over all particles within the domain after each time step. We reduce the smallest mesh size *h*_*m* *i* *n*_ over the whole domain after each time step, too. The time step size Δ*t* ^(*n* *e* *w*)^ then results from

$$\Delta t^{\left(new\right)}\leftarrow \min\left(C_{\Delta t}\cdot \Delta t^{\left(old\right)},\frac{h_{max}+2\varepsilon }{v_{\max}}\right)$$

with *C*_Δ*t*_ > 1. We typically employ *C*_Δ*t*_≈1.1.

A *veto mechanism* is able to cap the time step size from hereon: For each particle‐to‐particle comparison, the veto code studies those particle pairs that approach each other. For those, we distinguish a non‐critical from a critical approach. These studies focus on one‐dimensional setups. They study the particle behaviour along the distance between the two particle centres.

We first determine the space between the two particles' halo bounding spheres and draw a line from one centre to the other. The particles' velocities are projected onto this line. Their difference yields relative velocities between the particles. We next determine a maximum time step size along this line that would not make the two particle bounding spheres plus an *ϵ* environment overlap at the extrapolated positions of the next time step. If the determined maximum relative velocity times the given time step size is greater than the extrapolated remaining halo‐bounded distance, we label this situation as a critical approach. For a critical approach, the maximum time step size that is comfortable to the DEM simulator is smaller than the used time step Δ*t*. We restrict the admissible time step size of all critical approaches. Instead of a creeping increase, we then set *t* ^(*n* *e* *w*)^ to the smallest reduced time step. Any non‐critical approach has no further impact on the time step size choice.

Our approach is not arbitrarily robust. As we study only relative movements along lines between two particles and as we focus on two‐body problems, we assume that no rotation or impact from a third body might render our assumptions void. If large particles bump into very small particles, sudden accelerations of small particles however can still make these particles penetrate other particles. If particles are very largely and non‐symmetrically extended around the particle's centre of mass, then rotations also can lead to penetrations. An absolutely robust explicit scheme would have to rely on rollbacks of invalid solutions and reruns with smaller time steps.

For the present experiments, no penetration did occur for either strategy to determine Δ*t*. The experiments' objective is to demonstrate that the reluctant adaptivity criterion not only reduces the cell count of the adaptive grid. It notably facilitates bigger *h*_*m* *i* *n*_ over longer time spans for some simulations. Robustness studies and improvement strategies are beyond the scope of the present paper.

## 6 PARALLELISATION

### 6.1 Three layers of parallelism

Three layers of shared memory parallelisation arise naturally from our data structure choices. All three can be combined. They are conceptionally orthogonal to each other, though the efficiency of one might depend on choices made for the other. If they are combined, their results, ie, the bookkeeping of contact points, have to be protected by global semaphores. The identification of contact points however occurs infrequently compared to the triangle count. The total synchronisation penalty thus is negligible.

We run $O(T_{\max}^{2})$ triangle‐to‐triangle comparisons per particle pair if the particles approach each other and are reasonably close. These comparisons can be parallelised via a plain parallel for. Let $\left|T_{i}\right|$ and $\left|T_{j}\right|$ be the number of triangles of two particles *p*_*i*_ and *p*_*j*_. A collision detection between *p*_*i*_ and *p*_*j*_ has a concurrency level of $\left|T_{i}|\cdot |T_{j}\right|$. It is convenient for example to check the first triangle of particle *p*_*i*_ against all triangles from *p*_*j*_ whilst we run concurrently the checks for the second triangle of *p*_*i*_. We refer to this intra‐particles concurrency as triangle‐based parallelism. It arises if the two particles' boundary spheres augmented by *ε* each overlap. It is determined by the number of triangles per particle.

A second layer of concurrency, the *particle‐based parallelisation*, arises from the fact that multiple particles can be assigned to one vertex or two vertices connected by a cell may hold more than two particles in total. This statement includes virtual particles. If a vertex holds more than two particles, the collision checks between these particles can be realised. They are triggered when the vertex is read for the first time throughout a grid sweep. If a vertex holds more than one particle, we furthermore parallelise the comparison of particle pairs when we enter a cell and compare different vertices' particles with each other. The concurrency enters our pseudo code in Algorithm 1 within the statement contacts(localParticles) (line 23 in Algorithm 1). The concurrency is determined by the number of particles assembled within a cell.

Since our grid owns the particles and since we traverse the grid to trigger particle comparisons, it is an obvious choice to parallelise the grid traversal itself (*grid‐based parallelism*). The actual particle updates in Algorithm 1 are embarrassingly parallel w.r.t. the fine grid vertices. We can take all vertices (of any level/tree fragment) concurrently and update the velocities, positions and rotations of all of their particles. The actual particle‐grid maintenance exhibits a lower concurrency level however. A particle may move at most from one vertex's list into the list of a cell‐connected vertex. We realise these moves whilst we run through the cell. As the particle reassignments modify particle lists, we may not concurrently run the particle‐grid maintenance on two vertex‐connected cells. Instead, it is convenient to run through the cells of a grid level in a red‐black Gauß‐Seidel fashion. We end up with 2^*d*^ sweeps over the grid where no sweep does process any vertex‐connected cells. Such a traditional colouring where we process every second cell along each coordinate axis concurrently ensures that the re‐assignment of particles that have moved in the grid does not induce read‐write race conditions. All collision checks finally can be run in parallel as long as we again use a thread‐safe container to collect the collision points. The evaluation of the adaptivity criteria finally requires additional synchronisation or colouring again. It relies on restrictions and we thus have to ensure that no two children vertex properties are accumulated into their parent concurrently. These operations however are negligible in terms of computational cost.

If the mesh changes, we continue to traverse the grid serially until the mesh change has completed. We have to lift and drop particles, initialise data structures, allocate memory, update virtual particle links, and so forth. These are operations with many serialisation constraints or synchronise through operating system calls anyway. It is thus convenient to wait until the part of the mesh becomes stationary again, ie, to skip the parallel treatment of affected grid regions for one grid sweep.

### 6.2 Limitations and side effects

Triangle‐based parallelism is promising for detailed particle meshes. It however suffers from two properties. The high comparison workload arises infrequently. We use a grid to reduce the number of particle‐to‐particle studies. Per particle pair residing in neighbouring mesh cells, ie, per potential collision partners, we first check whether their bounding spheres do intersect and whether they do approach each other. Only if this is the case, we trigger the triangle‐to‐triangle comparisons and thus kick off high workload.

Particle‐based parallelisation suffers from the idea of multiscale adaptive meshes. The vision behind our spacetree construction is to obtain meshes that adapt to the accommodated particles and make us compare as few particles to each other as possible. As a result, the number of particles per vertex is typically very small, and the number of particles per cell, ie, per 2^*d*^ vertex constellations, is limited also. The situations seems to change for virtual particles. Still, we note that it is convenient per cell to build up particle comparison lists prior to triggering any actual comparison. That is, any cell has to ensure that a real particle is not compared multiple times against the same virtual particle. Therefore, the impact of particle‐based parallelism is limited. We have eliminated this concurrency by a sophisticated helper data structure.

Grid‐based parallelism performs poorly for DFS as there are only 3^*d*^ cells loaded per step down within the tree. With 2^*d*^ colouring, this gives a concurrency level that alters between eight and one. Techniques how to rewrite DFS automatically and on‐the‐fly into BFS are well‐known.^19^ We use them in our experiments. For trees that yield regular grids, at least in parts of the domain, this increases the concurrency level. The larger the grid, the less restricting the 2^*d*^ colouring for the particle management is relative to the workload. Still, our code has to preserve a temporal ordering between the resolution levels. To build up all virtual particle lists, coarse grid regions have to be handled prior to the finer mesh resolutions. Consequently, the more adaptive a grid the smaller the obtained concurrency level, whilst the coarser mesh levels suffer from limited concurrency anyway. The former property is rendered worse by the fact that many grid levels carry solely virtual particles whilst virtual‐to‐virtual checks are omitted. Inhomogeneous particle sizes or inhomogeneous particle distributions hence constrain the maximum concurrency induced by grid parallelisation.

Codes with plenty of branching, a high intensity of integer arithmetics, and indirect scattered memory accesses are notoriously ill‐suited for vectorisation. As we do not assume all particles to consist of the same number of triangles, particle‐based parallelism is thus not a fit for vectorisation. Grid‐based parallelism by construction does not fit. Triangle‐to‐triangle comparisons at first glance are not candidates either as their underlying geometric operations are determined by many if statements and branches. For any two triangles, we have to calculate the distance between the points, the edges, and the faces. All of these computations comprise if statements. The minimum of the outcomes finally determines the contact point if the resulting distance vector underruns our 2*ε* threshold.

In the work of Krestenitis et al,^9^ we propose a modification of these geometric checks. We cast the contact identification into a constrained minimisation problem. An iterative Newton solve searches for the minimum distance of two points along the two planes spanned by the two triangles. There is an admissibility constraint on the found points, ie, they have to be within the triangles on. These constraints are enforced by a penalty term scaled with a Lagrange parameter that is added to the minimisation. This approach is bare of if statements and thus vectorises excellently, ie, notably, once we hard‐code the number of Newton iterations and pipe multiple triangle pairs simultaneously through the chip. Obviously, such an approach is however not robust. It fails as soon as the underlying minimisation problem becomes ill‐conditioned, ie, when two triangles become “too parallel.” We thus propose to add a postprocessing step to the distance calculation. It kicks in if the minimisation residuals remain high after a prescribed number of Newton steps (typically 4 or 8), and makes the comparison fall back to the geometric checks. For most of triangle pairs, the Newton solver converges and yields high vectorisation efficiency. For few pairs, it does not succeed and is followed by a strongly branching code fragments which does not vectorise.

All excellent vectorisation efficiency from the work of Krestenitis et al^9^ relies on a decent number of triangle‐to‐triangle comparisons. If we decompose each particle's triangle set too aggressively to increase the concurrency level for our triangle‐based parallelism, we harm the vectorisation efficiency. As we want to exploit SIMD features, the triangle‐to‐triangle concurrency level already is “exploited” to a fair degree before we start to use it to keep cores busy. In practise, the pay‐off between parallelisation and vectorisation efficiency has to be studied carefully. For our setups, we did balance them empirically, as a systematic study is an endeavour beyond scope for the present paper. We conclude that the parallelisation strategy that is most promising is of limited availability.

The parallelisation strategies are studied by means of a manycore parallelisation in our manuscript. However, the grid‐based parallelisation paradigm applies to distributed memory environments also. The grid‐based parallelism describes a classic domain decomposition approach. If we process cells in parallel, we can cut the domain on each level into pieces and handle each piece on one distributed memory node. The mesh partitioning yields a particle distribution. To ensure that all contact points are found, each domain chunk has to be augmented by one ghost cell layer per grid level. The particles within the ghost cells are replicated. We obtain a multiscale overlapping domain decomposition. With such redundancy, we can, along subdomain boundaries, balance which ranks handle which comparison within the particle‐based approach. A discussion of the distributed particle administration including parallelised lifts and drops can be found in the work of Weinzierl et al.^33^

### 6.3 Grid traversals as producer‐consumer pattern in task‐based programming

A fundamental problem with grid‐based parallelism is that the computationally heavy triangle‐to‐triangle steps take turns with the computationally cheap grid traversal steps which furthermore are subject to additional colouring constraints, comprise many case distinctions, recursive function calls, and indirect memory accesses. The grid traversal steps tend to be solely bandwidth‐bound and do not yield lots of Flops per second. The approach and sphere overlap checks are cheap. For sustainable performance, one has to balance the two phases. In the best case, most cores are busy with actual triangle‐to‐triangle checks, heavily vectorised as discussed before, whilst the mesh continuously drips through the machine.

The temporal shift of DEM's workflow by half a grid sweep creates a tree traversal where the launch of the actual collision detection routines is the last step per geometric entity. Furthermore, the result of this step, ie, a befilled contact point set, is not required prior to the subsequent grid sweep. We thus propose to cast the actual triangle comparisons of two particles into a task and to make the grid traversal a task producer.

The traversal runs through the grid and keeps all particle‐to‐grid associations consistent. Moreover, it evaluates all refinement criteria and identifies potential collision candidate pairs, ie, pairs of particles which are close and approach. Still, it never triggers an actual particle‐to‐particle comparison. Instead, collision candidates are wrapped up into a task which we fire and forget for the time being, ie, the mesh traversal continues immediately.

Such an approach relies on task stealing^39^ to keep all cores that are not used by the mesh traversal busy. They grab the actual particle‐to‐particle comparison tasks, run them, and eventually bookkeep contact points. These cores act as task consumers. At the end of a grid traversal, the traversing core waits until all of the fired tasks have completed. Throughout the wait, it helps out on their progression. We remove the high number of synchronisation points that arise if the mesh traversal directly issues particle‐to‐particle comparisons and waits for their completion. Instead, we use one global synchronisation at the end of each grid sweep.

## 7 RESULTS

All of our experiments were run on an Intel E5‐2650V4 (Broadwell) node with 24 cores. The Broadwell is clocked at 2.4 GHz. To extrapolate our insights to upcoming manycore processor generations, we performed the largest runs on an Intel KNL (model 7210) running at 1.30 GHz with 64 cores also. Intel's 2017 C++ compiler translated all codes, and Intel's Threading Building Blocks (TBB)^39^ are used to parallelise them. TBB's task concept allows us to translate our producer‐consumer pattern into a working code straightforwardly. A comparison to OpenMP's task concept or other competitors is beyond scope. In line with the work of Krestenitis et al,^9^ all triangle‐to‐triangle code fragments are carefully vectorised. Here, we employ Intel‐specific pragmas.

### 7.1 Two particles with different grid types and spherical shapes

We start with experiments on two particles. The studies are conducted over the unit square and the two particles are placed at (0.20.20.2)^*T*^ and (0.80.80.8)^*T*^. Their velocities are (0.10.10.1)^*T*^ and ( − 0.1−0.1−0.1)^*T*^. They are set on collision course. We make them have different sizes, ie, a diameter of 0.02 vs. a diameter of 0.2.

Our first experiment compares the three different grid types and sticks to a spherical particle model. Whilst the sphere checks act as precheck for triangulated particles later on, they are preluded by a check whether two particles approach each other or move away from each other. If particles move away from each other, we do not even compute any sphere overlaps. Otherwise, we count the check as comparison here.

**Figure 3:**
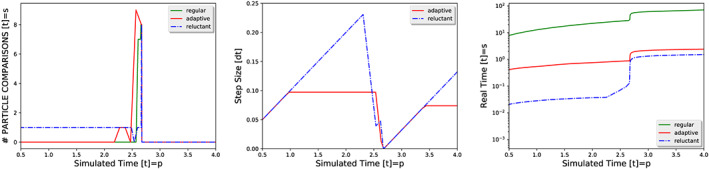
Zoom into collision behaviour for two particles and different grid types. We present number of collisions over simulated time (left). The time step sizes creeps toward the maximum time step size and then goes close to zero when the two fast particles collide (middle). Regular grids yield the same time step size pattern as the adaptive mesh. The compute time on a single core is given on the right for all three mesh choices

For a regular grid, the two particles live on different grids. Both particles move through their grid without any comparisons (Figure 3). The coarser particle induces virtual particles on the finer meshes, but these virtual particles do not induce comparisons in turn. When the two particles close up, virtual particles are inserted into vertices of the fine mesh which are cell‐connected to the anchor vertex of the real fine particle. All particle‐to‐particle comparisons then result from inter‐grid comparisons realised through particle‐to‐virtual particle checks. Once the two particles are very close, multiple comparisons are done as the bigger particle induces multiple virtual particles associated to many fine grid vertices. In an element‐wise traversal, only redundancies within the vertex and the cells can be identified and eliminated a priori, and we thus end up with up to 2^*d*^ = 8 comparisons of the particles. All remaining redundant contact points are eliminated in a postprocessing step. As soon as the spring dashpot potential manages to push the two particles away from each other, our precheck identifies that no contact point can arise and no comparisons are done anymore. The number of comparisons falls back to zero.

The adaptive grid yields a similar main spike of comparisons, which is preceded by few comparisons. These comparisons arise from hanging nodes which are temporarily created whilst the fine mesh region merges into the coarse grid region holding the coarse particles. To speed up all computations, we omit complicated geometric checks for hanging nodes, their creation tends to synchronise all computations already anyway, and make them simply link to all coarser vertices around through virtual particles. From these hanging nodes, we get, once throughout the simulation, one additional virtual particle from the coarse particle on top of the eight comparisons. Once the particles move away from each other, no comparisons are triggered anymore.

The reluctant grid yields a higher total comparison count. It starts from a rather coarse grid and triggers “unnecessary” comparisons. They identify that particles are approaching each other. For a substantial time, their bounding spheres however do not intersect. Once they are close enough, the grid eventually refines and we have few time steps without any collisions, before the real collisions yielding forces kick in. After the collision, no checks are triggered anymore and the mesh coarsens aggressively (not shown). Even if particles were represented by triangles, all of the unnecessary particle‐to‐particle comparisons would remain cheap as they immediately find out that the particles' bounding spheres do not overlap. The count numbers do not translate into runtime. Their studies and runtime measurements here provide pessimistic bounds or trends.

The adaptive grid outperforms the regular grid. Both allow the time step size to creep toward the maximum time step size enabled by the finer grid. Upon their close‐up, the time step veto mechanism dramatically shrinks the time step size. Afterwards, it “recovers.” All approaches reduce the time step size dramatically once they identify a (potential) collision, before they make it rise again. As they yield a similar timing pattern, it is the adaptivity's savings in terms of mesh cells that pays off here. Its mesh traversal is faster than the regular grid. We next compare the adaptive to the reluctant grid. In return for the higher number of checks, the reluctant grid is able to use wider time steps throughout the computation as it employs way coarser grids for most of the time. Only close to the actual collision, the veto mechanism reduces the admissible time step size “too” dramatically.

Still, the too pessimistic sudden drop does not cause harm in terms of runtime: The reluctant grid's bigger time step size makes up for the increased number of comparisons. Reluctant grids are the data structure of choice here. This variant only outperforms the adaptive grid if particles are reasonably away from each other, ie, not too densely packed. The denser the packing, the harsher the constrain on time step sizes and the fewer the opportunities for the aggressive coarsening to trigger mesh erases. For sparse particle populations, we have to assume that the performance gap furthermore closes once we use triangulated meshes and the cost of particle‐to‐particle comparisons start to dominate the runtime.

### 7.2 Two triangulated particles

We next examine the impact of the triangle‐to‐triangle comparisons. Excellent vectorisation has been shown before^9^ for reasonable triangle counts. Our present results stick to the two‐particle setup and they focus solely on the grid sweeps where actual triangle comparisons are to be made.

**Figure 4:**
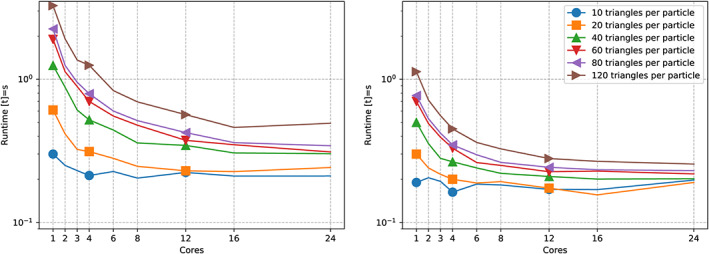
Zoom into collision behaviour for two particles discretised with different triangle per particle counts. We track the scalability of a non‐vectorised code (left) against a scalability graph for an executable with vectorisation and triangle‐based parallelism switched on (right)

We see a runtime improvement through vectorisation (Figure 4) of up to a factor of two, if a sufficient number of triangles (a few hundreds) is used per particle. Even though we have validated that the compiler does vectorise all triangle‐to‐triangle comparisons and does align all geometric data structures properly, this is disappointing. Obviously, larger triangle counts are required. In practise, this is not realistic. We are happy to work with dozens of triangles per particle. However, modern SIMD scatter and gather operations will allow us to pipe multiple particle comparisons through the chip in one rush. As clarified, the potential of particle‐based parallelism is limited, as there are only few comparisons to be made in parallel per cell or vertex, respectively. However, it is exactly this “few,” which might come in convenient for the vectorisation. We eventually will exploit the whole register width.

Unless we omit vectorisation, it does not make sense to exploit more than four cores solely for the comparisons. Beyond this, we enter the classic strong scaling regime and are not efficient anymore. For the end‐to‐end simulation, this four is even too optimistic, ie, only very few particle pairs actually become subject to triangle‐to‐triangle comparisons.

Our code benefits from aggressive vectorisation (AVX) of modern microprocessors and the collision prechecks with spheres and particle approaches as well as our sophisticated grid data structures minimising checks. Together, these properties imply that a very limited shared memory upscaling potential does exist on the triangle level. Scalability has to stem from other approaches. Yet, once two particles are compared, more than one core should be used. This calls for a lightweight parallelisation as it is offered through Intel's Threading Building Blocks which does not stationary assign compute resources to particular algorithmic steps. Indeed, we conducted experiments with TBB plus OpenMP on the triangle‐to‐triangle level and found the resulting performance of such a hybrid disappointing. The present data stem from TBB runs only.

### 7.3 Many‐particle systems

We conclude the experiments with a simulation of the hopper setup from Figure 2. A set of either 1,000 or 10,000 particles, arranged in a lattice layout, are dropped into a hopper geometry, squeeze through it, and fall on the floor below. All particles are represented by triangles. We construct them from spheres, which are triangulated and then the resulting vertices are dented in or extricated.

Hopper experiments serve as benchmarks for many DEM codes, since hoppers are used in many industrial workflows. Reasonable particle counts which yield insight on the flow behaviour range from 100,^23^ 800,^40^ over 3,125^10^ to 6,000.^41^ Experiments with spherical or analytical particle descriptions, which are not the focus of the present paper, in contrast work with 100,000 particles upwards^3^, ^5^, ^42^ and can even 2·10^9^ particles in total with more than 15,000 particles per rank/node.^1^ Our experimental setup thus covers the regime of the state‐of‐the‐art. Reports suggest that non‐spherical particle shapes, for these setups, alter the qualitative and quantitative behaviour of the overall system, ie, non‐spherical shapes increase the effective granular flow resistance and affect the piling behaviour at the hopper outflow.^10^, ^24^, ^26^, ^41^ Single node performance data for triangulated particles of inhomogeneous size however is, to the best of the authors' knowledge, not published yet.

**Figure 5:**
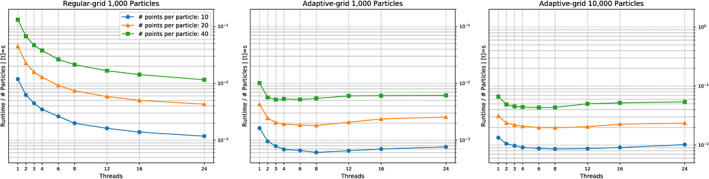
Scalability graph for a setup with different thread counts utilising a plain realisation of the grid‐based parallelism. We compare different grid types (left and middle) and particle counts (middle and right)

Experiments with plain grid‐based parallelism, all grid concurrency stems from standard colouring and additional concurrency levels, as discussed in the work of Krestenitis et al,^9^ are disabled and yield mixed results (Figure 5). Regular grids give us timings that are qualitatively almost independent of the triangle count. Furthermore, the scalability is limited. The triangle‐to‐triangle concurrency benefits from increased triangle per particle counts, but this improvement does not translate into the whole simulation. The improvements are eaten up by increased memory bus pressure created through many threads. Indeed, hardware performance counters suggest that the algorithm moves from a solely compute‐bound problem into a memory‐bound regime (not shown), and thus eventually exhibit triangle‐independent scaling.

Adaptive grids outperform their regular counterpart by one order of magnitude. However, they exhibit almost no scaling and a decrease of performance once we leave one socket of our two‐socket system. The larger the experimental setup the smoother all measurements. Still, scaling does not significantly improve. The reluctant grid (not shown) did not yield any different results from the adaptive grid. This behaviour is to be expected given the comparably dense packing of all particles.

Naïve mesh‐based and particle‐based parallelisation of our DEM code do not yield parallel efficiency computational scientists would like to see. Whilst adaptive and reluctant grids outperform their regular counterparts in time‐to‐solution, they make the scaling even worse. This is not a surprise, but results from the motivation of adaptivity: The grids ensure that not too many particles co‐exist within a cell, whilst the complicated Adaptive Mesh Refinement (AMR) multiscale structure introduces many synchronisation points and the colouring constrains the theoretical concurrency. We use techniques from the work of Weinzierl^19^ here, ie, we already transform DFS into BFS wherever this is appropriate. BFS' level‐wise processing yields higher concurrency under mesh colouring. Despite this advanced mesh reordering, the parallel efficiency remains limited. There are high concurrency phases, but they are interrupted by lots of tiny close‐to‐serial fractions where mesh refinement or adaptivity criteria evaluations kick in or the grid traversal switches from one level to the other.

**Figure 6:**
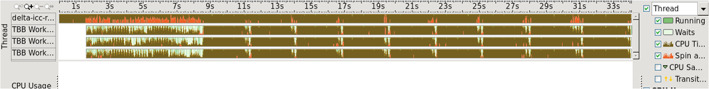
Screenshot of Intel's VTune validating that the task producer‐consumer pattern pays off after an initialisation phase of 9s where the grid is constructed and the particle shapes are built up

A producer‐consumer pattern changes these characteristics (Figure 6). It deploys the actual collision detections to tasks, which are stolen by idle threads, whilst the main thread continues its traverse. At the end of the traversal, the main thread joins its “worker” threads to complete all comparisons. A brief serial phase follows. Here, we remove duplicate contact points, derive the forces from the collisions, and kick off the subsequent mesh traversal. All worker threads briefly continue to idle until new collision tasks become available.

**Figure 7:**
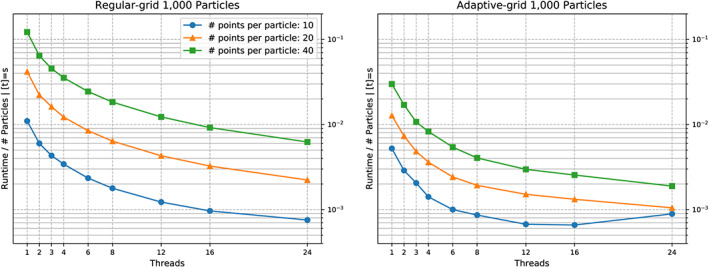
Experiments from Figure 5 rerun with a task‐based consumer‐producer realisation of the grid‐based parallelism

**Figure 8:**
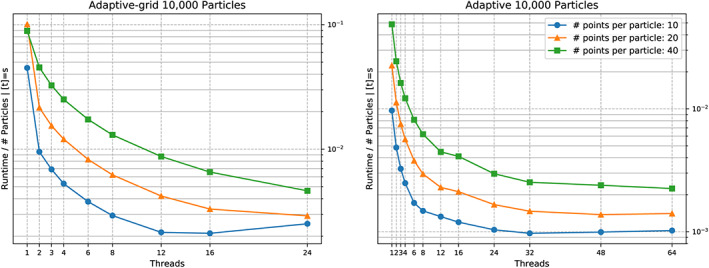
Experiments from Figure 7 with 10,000 particles on the Broadwell (left) and a KNL (right)

We finally observe that the task‐based realisation of the grid parallelism makes our algorithm scale reasonably (Figures 7 and 8). A task‐based formalism speeds up the treatment of regular grids, as it removes the synchronisation points when we switch from one level to another. Its full potential becomes apparent when we switch on the adaptivity. To stress the task formalism, these experiments also process the actual triangle‐to‐triangle comparisons concurrently.^9^ The actual comparisons thus pass through quickly. The tasks are small. The approach requires the task approach to scale. Although the actual particle‐to‐particle comparisons are few in an adaptive grid, weak scaling behaviour remains observable. The heavier the workload per particle pair (the higher the triangle count), the better the scaling. The task formalism brings together the advantages of adaptive grids with multithreading.

## 8 CONCLUSION AND OUTLOOK

We have proposed an organisational data structure that allows us to scale explicit DEM codes with global adaptive time stepping on a state‐of‐the‐art multicore processor. Together with the work of Krestenitis et al^9^ focusing on the vectorisation, it becomes clear that new hardware generations will eventually allow us to run decently sized DEM simulations with non‐analytical particles. Such simulations of real‐world setups are amongst our next steps, though it might be reasonable to first fuse our ideas with existing mature DEM codes offering a richer set of physics, experimental setups, postprocessing, and so forth.

On the methodology side, interesting follow‐up questions remain unanswered or did arise. On the one hand, we have to evaluate and understand the interplay of our techniques with time stepping in more detail. It is self‐evident that reluctant refinement and aggressive coarsening unfold tremendous power once they are combined with local timestepping, where particles advance in time with their own time step sizes. There also seems to be great potential for implicit time discretisations. DEM's locality property yields very sparse implicit time stepping matrices, which even become reducible if the time step sizes (slightly) underrun the time to collision between particles or particle clusters. The latter renders implicit solves without global communication possible. We again hypothesise that the choice of the right data structure and a tailored time stepping strategy on hierarchical data structures allows us to achieve such a decomposition effect on purpose. We might be able to make the grid refine such that implicit time stepping does not have to communicate globally.

A tailored performance‐aware adaptivity criterion also has to anticipate that it seems to be reasonable not to “over‐discretise” the mesh. Modern architectures are well‐suited for streaming data flow, and it might be advantageous to choose mesh cells bigger to keep the memory controllers busy and to allow gathering vectorisation to exploit SIMD. A combination of our cell approach with Verlet lists might be the method of choice.

On the other hand, there will be novel MPI challenges, ie, our paradigms translate to distributed memory environments, as they orbit around (multiscale) domain decompositions. The term domain for us comprises both mesh and particle data. How to load balance these domains plus how to realise the communication data flow remains open. There are at least two properties however that are very promising, ie, our algorithm spans halo layers hierarchically. It needs a halo layer on each and every resolution level. Data from these halo layers is not required to be all available at the same time, as our algorithm progresses from coarse to fine resolutions. Furthermore, the symmetry of the collision detection allows for strategies to keep halo layers not bit‐wisely consistent/redundant. Potential redundancies where two ranks would find the same collision point through the halo can be identified a priori and used to sparsify all data exchanged whilst data from coarse resolution levels is sent out prior to other data. Fine resolution data thus has more time to travel through the network and to hide behind computations.

For explicit time stepping with non‐volumetric particles of dramatically different speed, previous work in the Particle‐In‐Cell (PIC) context^33^ has shown that it is advantageous to analyse which reductions and data exchanges can be partially skipped throughout a time step. Hierarchical meshes help with this. Skips reduce the pressure on the communication network and they mitigate latency sensitivity and synchronisation penalties. Again, there seems to be great potential notably for local and implicit time stepping in the DEM context. Similar to the spawning of background tasks to compute collisions, it should be possible to identify data flow patterns prior to their realisation, to skip data exchange that reduces to sole synchronisation, and eventually even to tailor the adaptivity criteria such that they yield more of these skips.
